# The Week's Work

**Published:** 1910-01-01

**Authors:** 


### THE WEEK'S WORK.

### "DESICCATED RUBBISH."

THERE is an unfortunate prejudice in English hospitals against the use of dried vegetables. We have heard one authority condemn their use as unworthy of a modern hospital, and referring to them as desiccated rubbish which is not worth the trouble of preparing for the patients' consumption. One reason for this prejudice is undoubtedly the fact that green and fresh vegetables are nearly everywhere obtainable, and that it is regarded as essential that where it is possible to obtain such supplies it is necessary that they should be bought, since the health of the patients demands that they should be fed on fresh food. There can be little question, however, that fresh vegetables, even in country districts, are more expensive than dried, and from a dietetic point of view there is little to choose between the two forms. Æsthetically much may be said in favour of fresh vegetables, but it is difficult to see where the æsthetic question comes in where patients in a hospital ward are concerned. The vegetables are served out from the ward table, and each one gets his portion of cabbage or potatoes without being able to see the cabbage as a whole or revel in the sight of the unbroken green, which Brillat Savarin held was one of the pleasures of serving "formed" vegetable dishes instead of mushes. Certainly the æsthetic objection falls to the ground in the case of soups or stews. As a matter of fact, vegetable soups, which are now so largely used in Continental and American institutions, have not yet won popular favour in English hospitals, but they can be made from dried vegetables—for example, desiccated potatoes—just as well as from the green stuff.

### WARD COOKERY.

ANOTHER reason for the unfortunate prejudice against "desiccated rubbish" is the fact that the vegetable cookery in English hospitals leaves very much to be desired. Boiled cabbage as served at a ward dinner is about the most awful dish that misdirected culinary ingenuity can concoct. Boiled carrots, turnips, marrows, and artichokes (for some occult reason cucumbers, squashes, broad-beans, and spinach rarely figure in ward menus) are very little better, and the fact that boiled potatoes are so excellent in general is simply due to the fact that by steaming the most ferocious of hospital cooks can hardly damage the potato. In Continental hospitals vegetables appear under different guises—disguises our conservative ward cooks would term them—that enhance their merits as food and undoubtedly render them much more attractive to patients. We need only refer to the succulent *fritturas* of the Italian hospitals and to the stuffed vegetables that are so prominent a feature in German institutions. The fact is that dietetics as an art is hardly yet understood by our hospital cooks, and that the lessons in sick-room cookery, which every nurse ought to know, are not carried into practical use in the wards. Few physicians or surgeons concern themselves about what their patients eat. They are content to note that "full," "milk," or "thin" diet is adhered to, and they rarely, if ever, see that the full diet especially comes up to the standard they themselves would insist upon in their own homes. How many members of the staffs of our London hospitals, we wonder, know how a leg of mutton should be boiled? How many are content that their patients should get "boiled mutton" which has had all its nourishment and palatability taken out of it by being slowly heated up to boiling point in cold water?

### THE GERMAN EXAMPLE.

THESE reflections, however, are leading us astray from the main point, the employment of dried vegetables, which are largely used abroad and which readily lend themselves to the preparation of a variety of appetising and excellent dishes. In the current issue of the *Zeitschrift für Krankenanstalten* a hospital superintendent gives an account of his experiences with these preparations, which is eminently worth studying by those who take an interest in the institutional commissariat department. The writer prefers dried French beans, carrots, red and green cabbage, spinach, julienne mixed, and potatoes, and allows his patients an average of 30 grammes each of whatever vegetable figures on the daily menu—a quantity equivalent to 350 grammes of fresh green vegetables. The desiccated preparations are bought only from well-known makers; they are of recognised purity, but each lot is tested before acceptance, and must be up to the high standard fixed by the hospital board. A special dry room is used for the storing of the casks, which before being stored are allowed to remain in another chamber heated to 60° C. in order to free the dried preparations from any accidental moisture which they may have acquired during transport. Unless such care is taken in selecting and storing the desiccated vegetables they are apt to become mouldy, or at any rate musty. None of the preparations are used when fresh vegetables are cheap and plentiful—as in summer—but in winter their use is found to be economical. Dishes made from them are much appreciated by the patients, and can be prepared in a variety of ways, preferably as stews or vegetable soups. Properly selected preparations are free from all suspicion of mustiness, and preserve the flavour and relish of the fresh greens to a very large extent.

### WOMEN AS INFIRMARY DOCTORS.

CONSIDERABLE discussion has been aroused by the action of the committee of the Manchester Royal Infirmary in refusing to appoint women as medical officers of the institution. For a full report and the letters on the subject we refer readers to the *Manchester Guardian* of the 6th inst., in which several columns are devoted to the matter. A point was made of the alleged fact that the Royal Free Hospital, which was cited as a case of a mixed general hospital possessing women officers, was purely a women's hospital, having for its primary object the employment of medical women. Dr. F. Dickinson Berry has ably refuted that argument, showing that the Royal Free was originally a London general hospital without a medical school. "In 1877," Dr. Berry writes, "the wards were opened to the students of the London School of Medicine for Women. Since this date the connection between the school and hospital has grown continually closer, though the two still remain separate institutions. For many years women were not eligible for the resident posts, and students naturally felt that it was a great hardship, and also a handicap in regard to their future careers, that they could not hold these valuable appointments at their own hospital. About 1900 the committee of the hospital decided that two of the residents should be women, who would have charge of the female wards, and also take their share in the casualty work for both male and female cases. This arrangement has been carried out continuously since then, and I believe no one denies that it has worked satisfactorily. At the beginning of 1902, on the retirement of the physician for diseases of women, a woman was appointed to that post on the staff. Since then the number of out-patients has increased steadily."

### RESIDENT POSTS FOR MEDICAL WOMEN.

The committee appointed earlier in the year by the Infirmary to consider this question thinks it undesirable to appoint medical women to resident posts at the infirmary on the following grounds: "That in a general hospital the services of medical women are not, upon the whole, as valuable and beneficial as those of men. That such appointments would entail substantial building addition, and additions to, and a rearrangement of the staff, involving considerable expense, and that the Board would not be justified in incurring such expenses for the purpose of an experimental change which we are of opinion would not be of advantage to the institution." Now the first ground is one which is certainly debateable, if only because there has been little experience of women in resident posts. It is worth while pointing this out for the excellent reason that, though the *prima facie* arguments against the filling of resident posts by women are enormous, there is sure to be a battle over this question in the near future. The report of the Special Committee has, apparently, excited some sharp criticisms locally, and the *Manchester Guardian*, as a Liberal paper which has to weigh its words on the women suffrage question carefully, accused the committee of prejudice, alleging in substance that the plea of want of accommodation was a stalking horse. We believe, however, that the key to the committee's position is really to be found in the word "experimental." The committee has recognised that women can give at present no guarantee that, supposing resident posts were offered to them, they would be able to fulfil all the duties of that office. This being so, they can hardly quarrel that a new infirmary, which has only just finished the building, should immediately start off to alter its plan by providing unexpected accommodation. At present, however, there is "an average of less than two per annum" of female medical students admitted every year. This is the answer to the question at the moment: not enough women take up medicine at present to make the demand for their admission to medical posts a real one. When the numbers of women doctors increase, as they are likely to do, this question will be raised very heatedly, but it will supply its own answer. It is only when women practitioners are relatively common that it will be possible to find out whether or not they are as generally valuable as men. The way to find this out is to look at numbers of them in private practice. It is for them to prove their case, not for the hospitals.

### NURSES AS SURGEONS.

This matter is one which demands the serious attention of the profession, for otherwise it may do as much injury to the general practitioner as professional carelessness in the prescription of tabloids has brought about. The ignorance which exists in some circles on the subject of the practice of skin-grafting by nurses is illustrated by the letter of a matron of one of the greatest and oldest training schools, who writes: "It seems to be an absolutely unnecessary question to ask, is it desirable that nurses should learn and practise skin-grafting?" The extent to which the practice has grown in certain Poor Law infirmaries may be gathered from the following letter which we have received from the matron of one of the largest of these institutions: "With regard to the question on skin-grafting, I should say, certainly it is desirable that nurses should learn and practise it, provided they do it only when ordered by the doctor. Here we have large numbers of ulcered legs, and skin-grafting is done, in many cases with very good results, by the ward sister. She teaches the nurses, but the doctor always decides when the leg is ready for it. Of course, Reverdin's method is the only one done by nurses here. Thiersch's method is, I believe, never done except by a doctor, and generally under an anæsthetic. That is usually done to cover large surfaces, like severe burns. I do not approve of nurses taking the graftings from their own arms. Here they are taken from the patient's arm or upper part of the leg, and strict asepsis is observed." We direct the attention of our readers to this really important question, which merits the immediate consideration of the medical profession, and also of the authorities of nurse training schools throughout the country.

### THE DRUG MARKET.

The drug market has been inactive during the greater part of the week, owing to the Christmas holidays, and no changes in value of any importance have taken place. The reduction in the price of methylated spirit announced last week takes effect to-day, when the price per gallon will be 2s. 7d. in ten-gallon lots, and 2s. 5d. per gallon in thirty-gallon lots. The syndicate in Turkestan has now fixed the price of santonin at 34s. per lb., but higher prices are expected. The following drugs are likely to advance in price: Ergot, sassafras, senega, and damiana, the price of the last named being already abnormally high.

